# Patterns of substance use in people with severe mental illness: A case-control study in Ethiopia

**DOI:** 10.1371/journal.pone.0332107

**Published:** 2025-12-05

**Authors:** Melkam Alemayehu, Awoke Mihretu, Ruth Tsigebrhan, Manasi Sharma, Bizu Gelaye, Solomon Teferra

**Affiliations:** 1 Department of Psychiatry, School of Medicine, College of Health Sciences, Addis Ababa University, Addis Ababa, Ethiopia; 2 Department of Epidemiology, Harvard T. H. Chan School of Public Health, Boston, Massachusetts, United States of America; 3 Epidemiology Branch, Division of Population Health Research, Division of Intramural Research, Eunice Kennedy Shriver National Institute of Child Health and Human Development, Bethesda, Maryland, United States of America; Curtin University Bentley Campus: Curtin University, AUSTRALIA

## Abstract

**Background:**

Substance use disorders are increasingly prevalent in sub-Saharan African countries, with evident bidirectional risk for people with severe mental illness (SMI) resulting in more severe and chronic courses. This study examines the association between common substance use, including khat and alcohol, and SMI, comparing affected individuals to age and sex-matched controls in Ethiopia.

**Method:**

This study uses data collected as part of the NeuroGAP-Psychosis project, a large multi-country case-control genetics study. This case-control study consisted of 6,500 people with severe mental illness and age and sex-matched 6,500 controls in Ethiopia. Current substance use was defined as any use within the last three months, while current regular use was defined as at least weekly use within the previous three months. Descriptive statistics were used to determine the demographic distribution of participants, and logistic regression was used to estimate the association between substance use and severe mental illness.

**Results:**

Most cases were men (65.8%; n = 4,276) with a mean age of 36.9 (SD = 10.3) years, while the controls were 64.7% (n = 4,204) men with a mean age of 36.2 (SD = 10.9) years. Current use of tobacco, alcohol, khat, and cannabis was 15.1%, 17.2%, 20.4% and 1.0% among cases and 6.0%, 41.5%, 12.5%, and 0.8% among controls, respectively. The adjusted odds of regular tobacco and khat use were 4.8 (95% CI: 4.0, 5.9) and 1.6 (95% CI: 1.4, 1.8) times higher in cases than in controls, respectively. However, regular use of alcohol was 0.1 (95% CI: 0.1, 0.2) times lower among cases than among controls.

**Conclusions:**

Tobacco and khat use were higher among cases than controls, while alcohol and cannabis use were more prevalent in controls. The high prevalence of khat use is particularly problematic in people with SMI as it adversely affects the course and outcome of mental illness. Tobacco smoking also contributes to physical health morbidity and associated premature mortality. Therefore, evidence-based screening and treatment services are warranted to mitigate the harmful health outcomes of khat use and tobacco consumption among individuals with SMI.

## Introduction

Mental disorders pose a significant global health burden, accounting for 7–16% of the total disability-adjusted life years worldwide [1,2]. Severe mental illnesses (SMI) refers to chronic and disabling mental health conditions that include schizophrenia and bipolar disorder which profoundly contribute to this burden by impacting individuals’ thoughts, emotions, and behaviors, and often impairing their ability to function in daily life and relationships [3]. This burden is even greater in settings like Ethiopia, where mental disorders still receive limited attention from policymakers, despite accounting for more than 11% of the years lived with disability in the country [4]. However, the actual causes and risk factors of SMIs are not well understood. Epidemiological evidence suggest that genetics, structural and chemical changes in the brain, childhood trauma, and previous and active substance use may be possible risk factors [5,6]. Therefore, this study examines the relationship between SMI and common substance use such as alcohol, khat, tobacco, and cannabis, by comparing individuals with SMI to age-and sex-matched controls.

Substance use disorders (SUDs) are common and often comorbid with SMI, with the comorbidity reported to be between 30% and 50%, more than two times higher than the rate of SUDs in the general population [7]. Available evidence, largely from high-income countries, demonstrates substance use disorders as risk factors that worsen symptom prognosis, causing morbidity and premature mortality among people with SMI [6,8]. In addition, SUDs significantly impact the quality of life, economy, and social relationships of affected individuals and their family members [9]. The dual burden often leads to adverse health outcomes, including poor physical well-being, inadequate self-care practices, homelessness, and criminal justice system involvement, thereby increasing the burden on healthcare systems [10,11].

Mental health problems and substance use disorders have been on the rise in low-and middle-income countries (LMICs) [11]. A 2018 meta-analysis showed that the overall prevalence of substance use was around 40 percent in sub-Saharan African countries [12]. The predominant substances used in Eastern African countries, such as Ethiopia, which is the second most populous sub-Saharan African country, are alcohol, inhalants, and khat [12]. Alcohol and khat, in particular, have seen significant increases in usage, particularly among younger populations in recent years [13]. A few cross-sectional studies conducted in Ethiopia explored the magnitude of substance use comorbidity among patients with SMI. They found that the current prevalence of use varies from 25% to 40% [14–17], and dependence was reported in 10–25% [16,18,19]. The cross-sectional nature of the previous studies and the higher prevalence of both conditions in males and younger age groups make it difficult to understand the magnitude difference in substance use between people with SMI and the general population [20]. Thus, this study will examine the association of common substance use among people with SMI compared to their age and sex-matched controls in Ethiopia.

## Methods

### Study design and setting

Data were gathered as part of the Neuropsychiatric Genetics of African Populations-Psychosis (NeuroGAP-Psychosis) study, a large-scale genetics research project. This primary study was a matched case-control study design where cases were people with a diagnosis of psychosis disorder attending medical care, and controls were individuals visiting the hospital for any medical condition, those attending to others with a medical condition, students, or hospital staff without a diagnosis of psychosis. The study defined psychosis as having one of the following conditions: schizophrenia; schizoaffective disorder; bipolar disorder, mania not otherwise specified, or psychotic disorder not otherwise specified. Age groups and sex variables were used as matching criteria for cases and controls through group matching.

The parent NeuroGAP-Psychosis study aimed to identify genetic and environmental risk factors for psychotic disorders in four African countries: Ethiopia, Kenya, South Africa, and Uganda [21]. This study analyzed data from the Ethiopia site. Data were collected from four different collection sites in Ethiopia: 1) Amanuel Mental Specialized Hospital, 2) Tikur Anbessa Referral Hospital, 3) Zewditu Memorial Hospital, and 4) Jimma University Teaching Hospital. In this study, Amanuel Mental Specialized Hospital only recruited cases, Tikur Anbessa Hospital only recruited controls, while the other two sites recruited both cases and controls.

The three sites, Amanuel Mental Specialized Hospital, Zewditu Memorial Hospital, and Tikur Anbessa Specialized Hospital, are located in the capital city, Addis Ababa. The fourth site, Jimma University Teaching Hospital, is located in Jimma City, 352 kilometers southwest of Addis Ababa. Recruitment for the study lasted from 19/06/2018 to 30/03/2023.

### Study participants

The primary study recruited all participants from the hospital setting, using the following inclusion and exclusion criteria. The inclusion criteria for cases were adults 18 years or older with a diagnosis of schizophrenia spectrum disorder or bipolar disorder (i.e., schizophrenia; schizoaffective disorder, bipolar disorder, mania not otherwise specified; or psychotic disorder not otherwise specified) and could be inpatients or outpatients. Inclusion for controls were being 18 years or older, having no current or history of psychotic disorder, and not taking any psychiatric medication. Controls were individuals who were at the hospital at the time of recruitment, including those with any medical condition other than a psychotic disorder, as well as healthy controls who were attending to people with other medical conditions, students, or staff at the time of recruitment. Demonstrating the incapability to provide informed consent as determined by the University of California, San Diego Brief Assessment of Capacity to Consent (UBACC) [21] and being currently admitted or having acute medical care for alcohol or substance use disorder served as an exclusion criterion for both cases and controls [22]. All participants were required to be proficient in either Afan Oromo or Amharic, the languages in which the study was conducted.

### Data collection procedures and instruments

Data were collected by a mental health professional (MSc mental health workers and psychiatrists), who had undergone extensive training on all the instruments involved, in addition to receiving refresher training on a regular basis. Data were collected using a structured questionnaire designed using electronic data capture software “Research Electronic Data Capture (REDCap)” [22].

#### Diagnosis of SMI.

Case definition was based on clinical diagnosis on the chart. For the purposes of this analysis cases were grouped into a binary group: schizophrenia spectrum disorder (i.e. schizophrenia; schizoaffective disorder; or psychotic disorder not otherwise specified) and bipolar disorder (i.e bipolar disorder or mania not otherwise specified). However, relevant sections of the Mini International Neuropsychiatric Interview (MINI) version 7.0.2 were also administered using the MINI app to identify symptom types.

#### Sociodemographic information.

This includes self-reported questions like 1) Gender reported by participants as female or male; 2) Age which ranged from 18–84 years and was grouped into 18–29; 30–44; 45–59; and 60 + groups; 3) Marital status categorized as: never married; married or cohabitating; and separated, divorced, or widowed; 4) Living arrangement defined as: lives alone; lives with parental or spousal family; lives with relatives or friends; and 5) Education status grouped as: no formal education, primary level, secondary level, and college and beyond.

#### Substance use.

Substance use was defined as ever having used any of the substances assessed as part of an abbreviated Alcohol, Smoking, and Substance use Screening Test (ASSIST) questionnaire, which includes eight items and an additional question on khat—the most commonly used substance in Ethiopia. The substances assessed include lifetime and current use of tobacco products, khat, alcoholic beverages including home breweries, cannabis, cocaine, amphetamines type stimulants, inhalants, other over-the-counter medicines, sedatives or sleeping pills, hallucinogens, and opioids. Participants were also asked about any other substances consumed along with the frequency of use, defined as lifetime use, current use (use within the last three months), once or twice use within the last three months, monthly, weekly, and daily or almost daily use. The ASSIST screening tool was developed by the World health Organization (WHO) as part of a multi-country initiative. It comprises items that have been validated as reliable and feasible for international use with an internal consistency ranging between 0.8–0.9 for each domain [23,24], and the tool has been utilized multiple times among people with mental health problems in the Ethiopian context as well [25–27].

#### Current substance use.

Current substance use was defined as the use of any of the substances within the last three months from the date of the interview.

#### Irregular substance use.

Irregular substance use was based on the frequency of use, and it includes monthly, never or once, or twice within the last three months.

#### Regular substance use.

Regular use was defined to include at least weekly use based on the frequency of use.

### Data management and analysis

Frequencies, proportions, and summary statistics were computed to describe the study population and the prevalence of common substance use. Any significant association within groups were examined using bivariate analysis and outputs were presented using tables and graphs. Since a matched design was used, this study controlled for the matching factors in the logistic regression analysis without needing to use a matched analysis like conditional logistic regressions [28]. The analysis examined substance use outcomes, specifically, current substance use as well as regular and irregular use of individual substances, each modeled separately. No issues related to multicollinearity were observed based on the variance inflation factor test. Similarly, the discriminatory ability of the logistic regression model was assessed using the receiver operating characteristic (ROC) curve. The area under the ROC curve (AUC) was 0.72, indicating the model’s ability to reasonably distinguish between cases and controls.

### Ethical considerations

The research obtained ethical approvals from all sites involved, including Addis Ababa University College of Health Science’s Institutional Research Ethics Review Committee (Ref. No: 014/17/PSY), the Ethiopian National Research’s Ethics Review Committee (Ref. No: 3.10/14/2018) located at the Ministry of Science and Technology, and the Harvard T.H. Chan School of Public Health (#IRB17-0822). Since the study included vulnerable subjects, ability to provide informed consent was assessed for all potential participants using the the University of California, San Diego Brief Assessment of Capacity to Consent (UBACC) instrument. Once ensured, written informed consent was obtained from all participants, certifying voluntary participation. 

## Results

### Descriptive characteristics of participants

The characteristics of the study participants are summarized in Table 1. A total of 13,000 participants were included in this study with 6,500 cases and 6,500 controls. The case group included 4,276 male participants (65.8%), with a mean age of 36.9 years with a standard deviation (SD) of 10.3 years, while the control group comprised of 4,204 males (64.7%) with a mean age of 36.2 (SD = 10.9) years. More than half of the cases (56.3%) were never married/single, while 28.2% were married and 15.4% were divorced or separated. Among controls, 37.9% were never married/single, while 50.9% were married and 11.2% were divorced or separated. More than one-third of the cases had secondary education (36.7%), while 33% had primary education and 24.2% had a college degree or more. In controls 43.9% of participants reported having a college degree or more and 30.2% had a secondary level education and 23.5% had a primary level education. In addition, more than two-thirds of both cases and controls lived with parental or spousal families (72.8% vs 81.5%), respectively.

**Table 1:** Demographic characteristics of participants.

Variables	Case (6,500) %	Controls (6,500) %	P-value
Gender			
Male	65.8	64.7	0.185
Females	34.2	35.3	
Age			
18-29	25.5	26.9	0.302
30-44	51.6	50.6	
45-59	20.0	19.5	
60+	2.9	3.0	
Marital status			
Single/Never married	56.3	37.9	0.000
Married/ Cohabitating	28.2	50.9	
Separated/ Divorced/ Widowed	15.4	11.2	
Education			
No formal education	6.1	2.4	0.000
Primary level	33.0	23.5	
Secondary level	36.7	30.2	
College +	24.2	43.9	
Living arrangement			
Lives alone	11.7	13.7	0.000
Lives with parental family/ Spouses	72.8	81.5	
Lives with relatives/ Friends	15.5	4.8	
Diagnosis (for cases only)			
SSD	75.5	–	–
BP		–	
Inpatient/Outpatient (n = 5,658) (for cases only)	24.5		
Outpatient	87.8	–	–
Inpatient	12.2	–	

*SSD: Schizophrenia Spectrum Disorder; BP: Bipolar Disorder; P-Value from Chi-square test in bivariate analysis.*

The primary diagnosis of the cases was schizophrenia spectrum disorder (75.5%), while the remaining were diagnosed with bipolar disorder (24.5%). Of these, only 12.2% were recruited from inpatients, while the remaining were outpatients.

### Pattern of substance use

**Fig 1:**
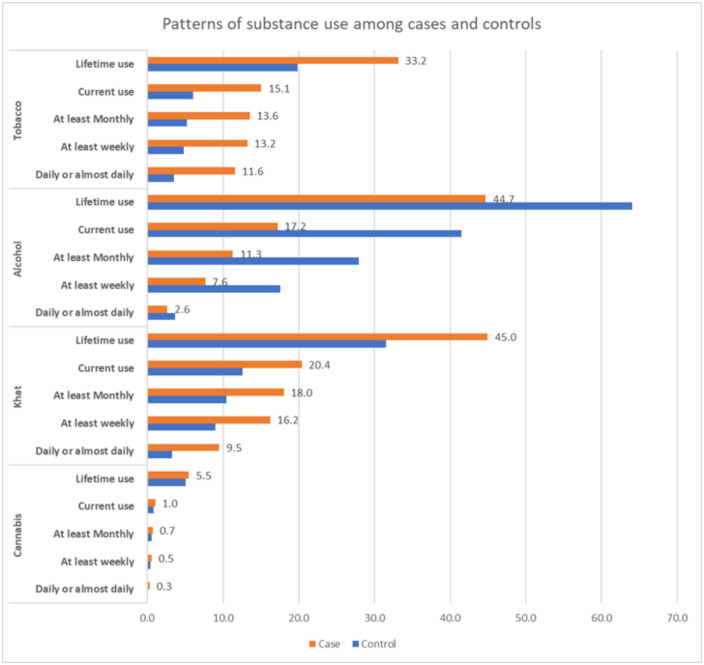
Patterns of substance use among cases and controls.

Substance use patterns are summarized in Fig 1 and Table 2. Alcohol and khat were the most common substances consumed among both cases and controls, with a proportion of 44.7% (95% CI: 43.5–45.9%) and 44.9% (95% CI:43.7–46.2%) for cases and 64.1% (95% CI: 62.9–65.2%) and 31.5% (95% CI: 30.4–32.7%) for controls respectively. Tobacco and cannabis use were 33.2% (95% CI: 32.0, 34.4%) and 19.9% (95% CI: 18.9–20.8%) for cases and 5.5% (95% CI: 4.9–6.1%) and 5.1% (95% CI: 4.6–5.6%) for controls, respectively. Current use of alcohol was higher among controls than cases: 41.5% (95% CI: 40.3–42.7%) vs. 17.2% (95% CI: 16.3–18.1%) respectively; whereas current use of other substances like khat and tobacco were higher among cases than controls (khat: 20.4% (95% CI: 19.4–21.4%) vs. 12.5% (95% CI:11.8–13.4%); and tobacco: 15.1% (95% CI: 14.2–15.9%) vs. 6.0% (95% CI: 5.5–6.6%)). However, cannabis use was similar across cases and controls: 1.0% for cases (95% CI: 0.8–1.3%) vs 0.8% for controls (95% CI: 0.6–1.1%) (Fig 1).

**Table 2:** Unadjusted and adjusted odds ratio (OR) and 95% confidence intervals (CI) for substance use in cases compared to controls.

Substance type	Frequency of use	Control (N = 6,500)		Case (N = 6,500)		Unadjusted OR (95% CI)	Adjusted OR (95% CI)
		N	%	N	%		
**Tobacco**	**Never used**	5,209	80.1	4,344	66.8	1.00 (Reference)	1.00 (Reference)
	**Lifetime use**	1,291	19.9	2,156	33.2	2.00 (1.85, 2.17)	2.38 (2.12, 2.67)
	**Irregular use**	978	15.0	1,297	19.9	1.59 (1.45, 1.74)	2.10 (1.85, 2.39)
	**Regular use**	313	4.8	859	13.2	3.29 (2.87, 3.77)	4.84 (3.98, 5.88)
**Alcohol**	**Never used**	2,337	35.9	3,593	55.3	1.00 (Reference)	1.00 (Reference)
	**Lifetime use**	4,163	64.0	2,907	44.7	0.45 (0.42, 0.49)	0.31 (0.29, 0.34)
	**Irregular use**	3,024	46.5	2,411	37.1	0.52 (0.48, 0.56)	0.38 (0.34, 0.41)
	**Regular use**	1,139	17.5	496	7.6	0.28 (0.25, 0.32)	0.12 (0.11, 0.14)
**Khat**	**Never used**	4,451	68.5	3,578	55.0	1.00 (Reference)	1.00 (Reference)
	**Lifetime use**	2,049	31.5	2,922	44.9	1.77 (1.65, 1.90)	1.63 (1.47, 1.81)
	**Irregular use**	1,467	22.6	1,867	28.7	1.58(1.46, 1.72)	1.65 (1.48, 1.85)
	**Regular use**	582	8.9	1,055	16.2	2.25 (2.02, 2.52)	1.56 (1.34, 1.81)
**Cannabis**	**Never used**	6,170	94.9	6,143	94.5	1.00 (Reference)	1.00 (Reference)
	**Lifetime use**	330	5.1	357	5.5	1.09 (0.93, 1.27)	0.69 (0.57, 0.82)
	**Irregular use**	305	4.7	322	4.9	1.06 (0.90, 1.24)	0.67 (0.55, 0.81)
	**Regular use**	25	0.4	35	0.5	1.41 (0.84, 2.35)	0.71 (0.39, 1.30)

*All variables are adjusted for Age, sex, marital status, living arrangement and education attainment as well as all the other substances

Similarly, khat was the most common regularly used substance reported by cases with 16.2% (95% CI: 15.3–17.1%), while alcohol was the commonly used substance reported by controls with 17.5% (95% CI:16.6–18.5%). Regular tobacco use was higher in cases than controls: 13.2% (95% CI: 12.4–14.1%) vs. 4.8% (95% CI: 4.3–5.3%) with a similar trend in daily use: 11.6% (95% CI: 10.8–12.4%) vs. 3.5% (95% CI:3.1–3.9%). Similarly, regular cannabis use was reported as 0.5% (95% CI: 0.4–0.7%) by cases and 0.4% (95% CI: 0.2–0.5%) by controls.

### Adjusted odds of substance use among cases and controls

After adjusting for the putative risk factors (i.e., age, sex, marital status, education, and living arrangement), khat and tobacco use were significantly higher among cases than controls. The odds of regular tobacco use were 4.8 (95% CI; 4.0–5.9) times higher among cases than controls, whereas irregular use was 2.1 (95% CI: 1.8–2.4) times higher. Similarly, regular use of khat was 1.6 (95% CI: 1.3, −1.8) times higher among cases than controls and irregular use was 1.6 (95% CI: 1.5, 1.8) times higher. Regular and irregular use of alcohol on the contrary was signifiacnlty lower (OR=0.1; 95% CI: 0.1, 0.2) and (OR=0.4; 95% CI: 0.3, 0.4), respectively, among cases compared to controls. However, regular use of cannabis was not significantly different among cases and controls while irregular use of cannabis use was lower (OR=0.7; 95% CI: 0.4, 0.8) among cases compared to controls (Table 2).

## Discussion

This study examines the association between SMI and the use of common substances—alcohol, khat, tobacco, and cannabis— by comparing individuals with SMI to age- and sex-matched controls. The study has three important findings. First, substance use was frequently reported among individuals with severe mental illness compared to controls. Of note, tobacco smoking and chewing khat were more than two times higher when compared to the controls. Second, current alcohol use was 70% lower among cases compared to controls. Lastly, no significant difference in regular cannabis use was found between cases and controls. However, irregular cannabis use was reported to be 70% lower among cases. This study highlights the association between commonly reported substances and individuals with and without SMI in Ethiopia. Such understanding can inform the design and implementation of effective intervention strategies in the country. Current tobacco use was three times higher among cases than controls, with 15.1% (95% CI; 14.2%, 15.9%) reported by cases and 6.0% (95% CI: 5.5%, 6.6%) by controls. Among female participants, the odds of current use was more than three times higher among cases, although the prevalence was notably low in both groups, with 1.5% of female cases and 0.4% of female controls. These findings align with previous studies conducted in Ethiopia and Nigeria [18,29]. Recent meta-analyses report a much higher pooled prevalence of tobacco among people with SMI [30,31]. However, these studies are primarily from high- and middle-income countries with higher national tobacco-use prevalence, such as China (26%), Japan (18%), the United States (13%), and Canada (12%) [32]. The national prevalence of tobacco in Ethiopia is approximatley 6%, which is similar to the prevalence among controls in this study [32,33]. The lower national prevalence might be explained by sociocultural and religious factors in this setting. The odds of tobacco smoking among cases is four times higher than controls. This finding is consistent with a well-established body of epidemiologic literature largely from the global north, showing that individuals with psychiatric disorders have a prevalence of tobacco use approximately two to four times higher than that of the general population [30,31,34].

Khat (Catha edulis) is a plant whose leaves and young buds are chewed to get a state of euphoria as a stimulant. The second most commonly used substance in Ethiopia, khat is usually chewed at social gatherings and is highly intertwined with cultural and religious beliefs. In the present study, the odds of khat chewing was almost two times higher in cases than in controls, with a lifetime prevalence of 45% among cases, consistent with other studies in similar settings [35,36]. This higher prevalence of khat use among cases might be explained by patients’ need to manage their symptoms, i.e., self-medication or tackle the side effects of the medications [37]. This is not surprising as the khat plant parts contain cathinone, an amphetamine-like substance, and small magnitudes of pharmacological compounds like *meru cathine, phenylpentenylamines and pseudomerucathine* [38]. Similar to other substance types and other study reports, khat use was more common among males, with only 3.3% of female cases and 2.4% of female controls reporting current use [35,39]. Cannabis was the least commonly reported substance, with a lifetime and current prevalence of 5.5% and 1.0% for cases and 5.0% and 0.8% for controls. There was no significant difference in regular cannabis use between cases and controls after adjusting for other variables; however, irregular cannabis use was 0.7 times lower among cases compared to controls. The overall reported magnitude of cannabis use was much lower compared to other similar studies [40,41], which might be attributable to the possible underreporting due to its illegal status in Ethiopia.

Regular and irregular use of alcohol were reported by 8% and 37% of cases and 17% and 46% of controls, respectively. In other words, the odds of drinking alcohol were 70% higher in controls than in cases. This finding is inconsistent with most [42–44], but not all, prior studies [42–45]. For instance, a large-scale study using the Genomic Psychiatry Cohort (GPC) reported that people with SMI reported higher alcohol use with an odds of 4.0 (95% CI 3.6, 4.4) compared to the general population [46]. Similarly, a systematic review and meta-analysis by Di Florio *et al*. also reported higher odds of alcohol use among people with bipolar disorder, along with significant heterogeneity influenced by factors such as geographical location and male-to-female ratio in the studies [42]. However, in agreement with this study, a study from South India by Kumar *et al*. reported the odds of alcohol use as three times higher in controls than cases [45]. Similarly, studies from Denmark and the United Kingdom found a higher prevalence of drinking in controls than cases [47,48]. This report of lower prevalence of alcohol consumption among cases might be explained by different reasons beyond geographical location. *First,* the reason for use of these substances may differ; patients with negative symptoms tend to prefer stimulants over substances with sedative effects, such as alcohol [49]. Even though further studies are required to validate this inconsistent findings across studies, the reported lower frequency of alcohol use might also be due to the difference in symptom level and symptom types of participants in the different studies. *Second,* another possible reason might be attributed to the financial demand that alcohol use requires compared to other common types of substances. Patients with SMI may already be financially disadvantaged because of their mental illness, potentially resulting in them not being able to spend as much when other common substances are available for a cheaper price [50]. *Third*, they may be likely to receive advice from health professionals and their caregivers against the use of alcohol, due to its interaction with psychiatric medications [51]. This may also increase the possibility of social desirability biases, resulting in underreporting by the patients.

Although this study examined the patterns of substance use among individuals with and without SMI in a largely representative sample across age groups and genders, certain study limitations should be noted. First, participants with acute or severe symptoms at the time of data collection were excluded, which may have led to an underestimation of the observed associations; however, due to the extended duration of the data collection, those with acute were more likely to be recontacted once they were in remission. Second, the self-reported nature of data collection may introduce recall or reporting bias, potentially resulting in underreporting. Third, the controls used for the study were not ideal controls, as they included hospital staff and students who were present at the hospital; moreover these controls were not entirely healthy controls.as such, the majority of the controls were individuals visiting the hospital for health conditions potentially influencing the reported substance use patterns, given the high comorbidity of substance use and chronic conditions [52]. Fourth, the primary aim of the study differed from the current objectives, which limited the assessment of certain potential confounders; however, this limitation may be partially mitigated by the large sample size included. Lastly, although the study grouped substance use into regular and irregular use, it was still measured based on solely on frequency, it did not assess the severity or apply diagnostic criteria for substance use disorder.

## Conclusion

In conclusion, the study found that tobacco and khat use were significantly higher among psychosis cases compared to controls, while no statistically significant difference was observed for regular cannabis use. However, alcohol and irregular cannabis use were more prevalent among controls than cases. Khat and cannabis are known to exacerbate existing severe mental illnesses, which adversely affect the course and outcome of the illness. Tobacco also contributes to higher physical morbidity and premature mortality in patients with severe mental illness. Therefore, patients need to be routinely screened for substance use, and early detection and integration of substance use treatment within the mental health care services are recommended. Further studies are needed to replicate these findings in similar research settings and confirm their generalizability. Additionally future researh should also examine the underlying motivations for substance use among these groups to support the development of targeted interventions that address the issue at its source.

## Supporting information

S1 Table Fully adjusted effects of substances use and socio-demographic characteristics on the odds of being a case versus a control. (DOCX)

## References

[1] Vigo D, Jones L, Atun R, Thornicroft G. The true global disease burden of mental illness: still elusive. Lancet Psychiatry. 2022;9(2):98–100. doi: 10.1016/S2215-0366(22)00002-5 35026138

[2] Whiteford HA, Degenhardt L, Rehm J, Baxter AJ, Ferrari AJ, Erskine HE, et al. Global burden of disease attributable to mental and substance use disorders: findings from the Global Burden of Disease Study 2010. Lancet. 2013;382(9904):1575–86. doi: 10.1016/S0140-6736(13)61611-6 23993280

[3] Killaspy H, White S, Lalvani N, Berg R, Thachil A, Kallumpuram S, et al. The impact of psychosis on social inclusion and associated factors. Int J Soc Psychiatry. 2014;60(2):148–54. doi: 10.1177/0020764012471918 23399990 PMC4107835

[4] IHME. GBD Compare: Ethiopia. Seattle, WA: IHME, University of Washington; 2019.

[5] van de Leemput J, Hess JL, Glatt SJ, Tsuang MT. Genetics of schizophrenia: historical insights and prevailing evidence. Adv Genet. 2016;96:99–141. doi: 10.1016/bs.adgen.2016.08.001 27968732

[6] Stilo SA, Murray RM. Non-genetic factors in schizophrenia. Curr Psychiatry Rep. 2019;21(10):100. doi: 10.1007/s11920-019-1091-3 31522306 PMC6745031

[7] Regier DA, Farmer ME, Rae DS, Locke BZ, Keith SJ, Judd LL, et al. Comorbidity of mental disorders with alcohol and other drug abuse. Results from the epidemiologic catchment area (ECA) study. JAMA. 1990;264(19):2511–8. doi: 10.1001/jama.1990.03450190043026 2232018

[8] Löhrs L, Hasan A. Risk factors for the development of schizophrenia. Fortschr Neurol Psychiatr. 2019;87(2):133–43. doi: 10.1055/a-0836-7839 30802921

[9] Daley DC. Family and social aspects of substance use disorders and treatment. J Food Drug Anal. 2013;21(4):S73–6. doi: 10.1016/j.jfda.2013.09.038 25214748 PMC4158844

[10] Donoghue K, Doody GA. Effect of illegal substance use on cognitive function in individuals with a psychotic disorder: a review and meta-analysis. Neuropsychology. 2012;26(6):785–801. doi: 10.1037/a0029685 22924618

[11] Uchtenhagen A. Substance use problems in developing countries. Bull World Health Organ. 2004;82(9):641. 15628199 PMC2622977

[12] Olawole-Isaac A, Ogundipe O, Amoo EO, Adeloye DO. Substance use among adolescents in sub-Saharan Africa: a systematic review and meta-analysis. S Afr J CH. 2018;12(2b):79. doi: 10.7196/sajch.2018.v12i2b.1524

[13] Fekadu AA, Charlotte H. Alcohol and drug abuse in Ethiopia: past, present and future. Afr J Drug & Alcohol Studies. 2007;6(1):14.

[14] Desalegn D, Girma S, Abdeta T. Quality of life and its association with current substance use, medication non-adherence and clinical factors of people with schizophrenia in Southwest Ethiopia: a hospital-based cross-sectional study. Health Qual Life Outcomes. 2020;18(1):82. doi: 10.1186/s12955-020-01340-0 32228624 PMC7106632

[15] Molla Z, Dube L, Krahl W, Soboka M. Tobacco dependence among people with mental illness: a facility-based cross sectional study from Southwest Ethiopia. BMC Res Notes. 2017;10(1):289. doi: 10.1186/s13104-017-2608-7 28716128 PMC5512937

[16] Tadesse H, Mirkana Y, Misgana T. Alcohol use disorder and its determinant factors among patients with schizophrenia attending treatment at mental specialized hospital, Addis Ababa, Ethiopia: a cross-sectional study. SAGE Open Med. 2021;9:20503121211048748. doi: 10.1177/20503121211048748 34603729 PMC8481719

[17] Ng LC, Medhin G, Hanlon C, Fekadu A. Trauma exposure, depression, suicidal ideation, and alcohol use in people with severe mental disorder in Ethiopia. Soc Psychiatry Psychiatr Epidemiol. 2019;54(7):835–42. doi: 10.1007/s00127-019-01673-2 30788553 PMC7343339

[18] Desalegn D, Abdu Z, Hajure M. Prevalence of tobacco dependence and associated factors among patients with schizophrenia attending their treatments at southwest Ethiopia; hospital-based cross-sectional study. PLoS One. 2021;16(12):e0261154. doi: 10.1371/journal.pone.0261154 34910737 PMC8673664

[19] Hailemariam M, Fekadu A, Medhin G, Prince M, Hanlon C. Equitable access to mental healthcare integrated in primary care for people with severe mental disorders in rural Ethiopia: a community-based cross-sectional study. Int J Ment Health Syst. 2019;13:78. doi: 10.1186/s13033-019-0332-5 31890003 PMC6935213

[20] Buckley PF. Prevalence and consequences of the dual diagnosis of substance abuse and severe mental illness. J Clin Psychiatry. 2006;67 Suppl 7:5–9. 16961418

[21] Stevenson A, Akena D, Stroud RE, Atwoli L, Campbell MM, Chibnik LB, et al. Neuropsychiatric Genetics of African populations-psychosis (NeuroGAP-Psychosis): a case-control study protocol and GWAS in Ethiopia, Kenya, South Africa and Uganda. BMJ Open. 2019;9(2):e025469. doi: 10.1136/bmjopen-2018-025469 30782936 PMC6377543

[22] Harris PA, Taylor R, Minor BL, Elliott V, Fernandez M, O’Neal L, et al. The REDCap consortium: building an international community of software platform partners. J Biomed Inform. 2019;95:103208. doi: 10.1016/j.jbi.2019.103208 31078660 PMC7254481

[23] WHO ASSIST Working Group. The alcohol, smoking and substance involvement screening test (ASSIST): development, reliability and feasibility. Addiction. 2002;97(9):1183–94. doi: 10.1046/j.1360-0443.2002.00185.x 12199834

[24] Humeniuk R, Ali R, Babor TF, Farrell M, Formigoni ML, Jittiwutikarn J, et al. Validation of the alcohol, smoking and substance involvement screening test (ASSIST). Addiction. 2008;103(6):1039–47. doi: 10.1111/j.1360-0443.2007.02114.x 18373724

[25] Mokona H, Yohannes K, Ayano G. Youth unemployment and mental health: prevalence and associated factors of depression among unemployed young adults in Gedeo zone, Southern Ethiopia. Int J Ment Health Syst. 2020;14:61. doi: 10.1186/s13033-020-00395-2 32782471 PMC7414568

[26] Wolde A. The relationship between khat use disorder and post-traumatic stress disorder among prisoners with life time trauma exposure in Ethiopia: a cross-sectional study. Neuropsychiatr Dis Treat. 2021;17:3669–81. doi: 10.2147/NDT.S336877 34934320 PMC8684415

[27] Tamene FB, Sema FD, Mihiretie EA, Siyum TS, Sendekie AK. Health-related quality of life and associated factors among patients with schizophrenia at comprehensive specialised hospitals in the Northwest Ethiopia: a multicentre cross-sectional study. BMJ Open. 2023;13(11):e074112. doi: 10.1136/bmjopen-2023-074112 37967996 PMC10660835

[28] Pearce N. Analysis of matched case-control studies. BMJ. 2016;352:i969. doi: 10.1136/bmj.i969 26916049 PMC4770817

[29] Aguocha CM, Aguocha JK, Igwe M, Uwakwe RU, Onyeama GM. Prevalence and correlates of cigarette smoking among patients with schizophrenia in southeast Nigeria. Acta Psychiatr Scand. 2015;131(3):206–12. doi: 10.1111/acps.12334 25209175

[30] Gurillo P, Jauhar S, Murray RM, MacCabe JH. Does tobacco use cause psychosis? Systematic review and meta-analysis. Lancet Psychiatry. 2015;2(8):718–25. doi: 10.1016/S2215-0366(15)00152-2 26249303 PMC4698800

[31] Ohi K, Shimada T, Kuwata A, Kataoka Y, Okubo H, Kimura K, et al. Smoking rates and number of cigarettes smoked per day in schizophrenia: a large cohort meta-analysis in a Japanese population. Int J Neuropsychopharmacol. 2019;22(1):19–27. doi: 10.1093/ijnp/pyy061 30239793 PMC6313124

[32] American Cancer Society, Vital Strategies. The tobacco atlas. Accessed 2025 July 10. https://tobaccoatlas.org/about/

[33] World Health Organization. WHO report on the global tobacco epidemic 2023: protect people from tobacco smoke. Geneva: World Health Organization; 2023. https://www.who.int/publications/i/item/9789240075793

[34] Lopes J, Silva I. Prevalence of tobacco smoking in patients with schizophrenia and bipolar disorder in a portuguese hospital. Acta Med Port. 2021;34(4):321–2. doi: 10.20344/amp.15909 34214427

[35] Abraha Gosh Woldemariam GT-T, Misgna Teklay Gebremedihin C. Catha edulis (Khat) use and demographic correlates of schizophrenia: a case-control study. Mental Health Science. 2023.

[36] Tulloch AD, Frayn E, Craig TKJ, Nicholson TRJ. Khat use among Somali mental health service users in South London. Soc Psychiatry Psychiatr Epidemiol. 2012;47(10):1649–56. doi: 10.1007/s00127-011-0471-8 22249804

[37] Teferra S, Hanlon C, Alem A, Jacobsson L, Shibre T. Khat chewing in persons with severe mental illness in Ethiopia: a qualitative study exploring perspectives of patients and caregivers. Transcult Psychiatry. 2011;48(4):455–72. doi: 10.1177/1363461511408494 21911510

[38] Graziani M, Milella MS, Nencini P. Khat chewing from the pharmacological point of view: an update. Subst Use Misuse. 2008;43(6):762–83. doi: 10.1080/10826080701738992 18473221

[39] Gebrehanna EBY, Worku A. Prevalence and predictors of harmful khat use among university students in Ethiopia. Subst Abuse. 2014;6. 10.4137/SART.S14413 PMC4055411 24940069

[40] Ayano G. Co-occurring medical and substance use disorders in patients with schizophrenia: a systematic review. Inter J Mental Health. 2019;48(1):62–76. doi: 10.1080/00207411.2019.1581047

[41] Pinto JV, Medeiros LS, Santana da Rosa G, Santana de Oliveira CE, Crippa JA de S, Passos IC, et al. The prevalence and clinical correlates of cannabis use and cannabis use disorder among patients with bipolar disorder: a systematic review with meta-analysis and meta-regression. Neurosci Biobehav Rev. 2019;101:78–84. doi: 10.1016/j.neubiorev.2019.04.004 30974123

[42] Di Florio A, Craddock N, van den Bree M. Alcohol misuse in bipolar disorder. A systematic review and meta-analysis of comorbidity rates. Eur Psychiatry. 2014;29(3):117–24. doi: 10.1016/j.eurpsy.2013.07.004 24075633

[43] Hunt GE, Large MM, Cleary M, Lai HMX, Saunders JB. Prevalence of comorbid substance use in schizophrenia spectrum disorders in community and clinical settings, 1990-2017: systematic review and meta-analysis. Drug and Alcohol Depend. 2018;191:234–58. 10.1016/j.drugalcdep.2018.07.011 30153606

[44] Koskinen J, Löhönen J, Koponen H, Isohanni M, Miettunen J. Prevalence of alcohol use disorders in schizophrenia--a systematic review and meta-analysis. Acta Psychiatr Scand. 2009;120(2):85–96. doi: 10.1111/j.1600-0447.2009.01385.x 19374633

[45] Kumar CN, Thirthalli J, Suresha KK, Arunachala U, Gangadhar BN. Alcohol use disorders in patients with schizophrenia: comparative study with general population controls. Addict Behav. 2015;45:22–5. doi: 10.1016/j.addbeh.2015.01.009 25634440

[46] Hartz SM, Pato CN, Medeiros H, Cavazos-Rehg P, Sobell JL, Knowles JA, et al. Comorbidity of severe psychotic disorders with measures of substance use. JAMA Psych. 2014;71(3):248–54. doi: 10.1001/jamapsychiatry.2013.3726 24382686 PMC4060740

[47] Schneier FR, Siris SG. A review of psychoactive substance use and abuse in schizophrenia. Patterns of drug choice. J Nerv Ment Dis. 1987;175(11):641–52. doi: 10.1097/00005053-198711000-00001 3316490

[48] Bernadt MW, Murray RM. Psychiatric disorder, drinking and alcoholism: what are the links?. Br J Psychiatry. 1986;148:393–400. doi: 10.1192/bjp.148.4.393 3730705

[49] Warner R, Taylor D, Wright J, Sloat A, Springett G, Arnold S, et al. Substance use among the mentally ill: prevalence, reasons for use, and effects on illness. Am J Orthopsychiatry. 1994;64(1):30–9. doi: 10.1037/h0079489 8147425

[50] Chong HY, Teoh SL, Wu DB-C, Kotirum S, Chiou C-F, Chaiyakunapruk N. Global economic burden of schizophrenia: a systematic review. Neuropsychiatr Dis Treat. 2016;12:357–73. doi: 10.2147/NDT.S96649 26937191 PMC4762470

[51] Weitzman ER, Magane KM, Wisk LE, Allario J, Harstad E, Levy S. Alcohol use and alcohol-interactive medications among medically vulnerable youth. Pediatrics. 2018;142(4):e20174026. doi: 10.1542/peds.2017-4026 30228168 PMC6317570

[52] Timko C, Kong C, Vittorio L, Cucciare MA. Screening and brief intervention for unhealthy substance use in patients with chronic medical conditions: a systematic review. J Clin Nurs. 2016;25(21–22):3131–43. doi: 10.1111/jocn.13244 27140392 PMC6430571

