# Supplementary material for: Patterns of substance use in people with severe mental illness: A case-control study in Ethiopia
Source: PLoS One. 2025 Dec 5;20(12):e0332107. doi: 10.1371/journal.pone.0332107 (PMC12680152; doi:10.1371/journal.pone.0332107)
Supplement: S1 Table — (DOCX) [file pone.0332107.s001.docx]

**Table 1: Fully adjusted effects of substances use and socio-demographic characteristics on the odds of being a case versus a control.**

| **Case/controls status** | **Cases**  **(6,500)**  **%** | **Controls**  **(6,500)**  **%** | **Unadjusted OR (95% CI)** | **Adjusted**  **OR (95% CI)** |  |  |  |
| --- | --- | --- | --- | --- | --- | --- | --- |
| Sex |  |  |  |  | |  |  |
| Female | 65.8 | 64.7 | 1.00 | 1.00 | |  |  |
| Male | 34.2 | 35.3 | 1.05 (0.98, 1.13) | 1.02(0.91, 1.12) | |  |  |
| Age |  |  |  |  | |  |  |
| 18-29 | 25.5 | 26.9 | 1.00 | 1.00 | |  |  |
| 30-44 | 51.6 | 50.6 | 1.08 (0.99, 1.17) | 1.65 (1.49, 1.83) | |  |  |
| 45-59 | 20.0 | 19.5 | 1.08 (0.98, 1.20) | 1.91 (1.67, 2.18) | |  |  |
| 60+ | 2.9 | 3.0 | 1.02 (0.83, 1.26) | 1.81 (1.41, 2.33) | |  |  |
| Marital Status |  |  |  |  | |  |  |
| Single/Never married | 56.3 | 37.9 | 1.00 | 1.00 | |  |  |
| Married/ Cohabitating | 28.2 | 50.9 | 0.37 (0.34, 0.40) | 0.24 (0.22, 0.27) | |  |  |
| Separated / Divorced/ Widowed | 15.4 | 11.2 | 0.93 (0.83, 1.03) | 0.63 (0.55, 0.72) | |  |  |
| Education |  |  |  |  | |  |  |
| No formal education | 6.1 | 2.4 | 1.00 | 1.00 | |  |  |
| Primary level | 33.0 | 23.5 | 0.55 (0.45, 0.67) | 0.45 (0.36, 0.56) | |  |  |
| Secondary level | 36.7 | 30.2 | 0.48 (0.39, 0.58) | 0.34 (0.27, 0.42) | |  |  |
| College + | 24.2 | 43.9 | 0.22 (0.18, 0.26) | 0.17 (0.13, 0.21) | |  |  |
| Living arrangement |  |  |  |  | |  |  |
| Lives alone | 11.7 | 13.7 | 1.00 | 1.00 | |  |  |
| Lives with parental family/ Spouses | 72.8 | 81.5 | 1.05 (0.94, 1.16) | 1.72 (1.52, 1.95) | |  |  |
| Lives with relatives/ Friends | 15.5 | 4.8 | 3.81 (3.24, 4.47) | 3.28 (2.96, 3.90) | |  |  |
| Tobacco use |  |  |  |  | |  |  |
| Never | 66.8 | 80.1 | 1.00 | 1.00 (Reference) | |  |  |
| Lifetime use | 33.2 | 19.9 | 2.00 (1.85, 2.17) | 2.38(2.12, 2.67) | |  |  |
| Tobacco regular and irregular use ^£^ |  |  |  |  | |  |  |
| Never use | 66.8 | 80.1 | 1.00 | 1.00 | |  |  |
| Irregular use | 19.9 | 15.1 | 1.59 (1.45, 1.74) | 2.10 (1.85, 2.39) | |  |  |
| Regular use | 13.2 | 4.8 | 3.29 (2.87, 3.77) | 4.84 (3.98, 5.88) | |  |  |
| Alcohol use |  |  |  |  | |  |  |
| Never | 55.3 | 35.9 | 1.00 (Reference) | 1.00 | |  |  |
| Lifetime use | 44.7 | 64.1 | 0.45 (0.42, 0.49) | 0.31 (0.29, 0.34) | |  |  |
| Alcohol regular and irregular use ^£^ |  |  |  |  | |  |  |
| Never use | 55.3 | 35.9 | 1.00 | 1.00 | |  |  |
| Irregular use | 37.1 | 46.6 | 0.52 (0.48, 0.56) | 0.38 (0.34, 0.41) | |  |  |
| Regular use | 7.6 | 17.5 | 0.28 (0.25, 0.32) | 0.12 (0.11, 0.14) | |  |  |
| Khat use |  |  |  |  | |  |  |
| Never | 55.1 | 68.5 | 1.00 (Reference) | 1.00 (Reference) | |  |  |
| Lifetime use | 44.9 | 31.5 | 1.77 (1.65, 1.90) | 1.63 (1.47, 1.81) | |  |  |
| Khat regular and irregular use ^£^ |  |  |  |  | |  |  |
| Never use | 55.1 | 68.5 | 1.00 | 1.00 | |  |  |
| Irregular use | 28.7 | 22.6 | 1.58(1.46, 1.72) | 1.65 (1.48, 1.85) | |  |  |
| Regular use | 16.2 | 8.9 | 2.25 (2.02, 2.52) | 1.56 (1.34, 1.81) | |  |  |
| Cannabis use |  |  |  |  | |  |  |
| Never | 94.5 | 94.9 | 1.00 (Reference) | 1.00 (Reference) | |  |  |
| Lifetime use | 5.5 | 5.1 | 1.09 (0.93, 1.27) | 0.69 (0.57, 0.82) | |  |  |
| Cannabis regular and irregular use ^£^ |  |  |  |  | |  |  |
| Never use | 94.5 | 94.9 | 1.00 | 1.00 | |  |  |
| Irregular use | 4.9 | 4.7 | 1.06 (0.90, 1.24) | 0.67 (0.55, 0.81) | |  |  |
| Regular use | 0.5 | 0.4 | 1.41 (0.84, 2.35) | 0.71 (0.39, 1.30) | |  |  |

** All effects reported in the table are adjusted for each other and also for each substance (i.e. for lifetime use of each substance)

# £ = The effect of lifetime use of each substance was not adjusted for its corresponding regular and irregular use due to multi-collinearity. However, it was adjusted for all variables listed in the table. (e.g. effect of lifetime tobacco use was not adjusted for regular and irregular tobacco use, and the same approach was applied for alcohol, khat and cannabis)
